# In pursuit of rigour and accountability in participatory design^☆^

**DOI:** 10.1016/j.ijhcs.2014.09.004

**Published:** 2015-02

**Authors:** Christopher Frauenberger, Judith Good, Geraldine Fitzpatrick, Ole Sejer Iversen

**Affiliations:** a Human Computer Interaction Group, Vienna University of Technology, Vienna, Austria; b Human Centred Technology Group, University of Sussex, Brighton, UK; c Participatory IT Center, Aarhus University, Aarhus, Denmark

**Keywords:** Participatory design, Reflective design, Rigour, Accountability

## Abstract

The field of Participatory Design (PD) has greatly diversified and we see a broad spectrum of approaches and methodologies emerging. However, to foster its role in designing future interactive technologies, a discussion about accountability and rigour across this spectrum is needed. Rejecting the traditional, positivistic framework, we take inspiration from related fields such as Design Research and Action Research to develop interpretations of these concepts that are rooted in PD׳s own belief system. We argue that unlike in other fields, accountability and rigour are nuanced concepts that are delivered through debate, critique and reflection. A key prerequisite for having such debates is the availability of a language that allows designers, researchers and practitioners to construct solid arguments about the appropriateness of their stances, choices and judgements.

To this end, we propose a “tool-to-think-with” that provides such a language by guiding designers, researchers and practitioners through a process of systematic reflection and critical analysis. The tool proposes four lenses to critically reflect on the nature of a PD effort: *epistemology*, *values*, *stakeholders* and *outcomes*. In a subsequent step, the *coherence* between the revealed features is analysed and shows whether they pull the project in the same direction or work against each other. Regardless of the flavour of PD, we argue that this *coherence* of features indicates the level of internal rigour of PD work and that the process of reflection and analysis provides the language to argue for it. We envision our tool to be useful at all stages of PD work: in the planning phase, as part of a reflective practice during the work, and as a means to construct knowledge and advance the field after the fact. We ground our theoretical discussions in a specific PD experience, the ECHOES project, to motivate the tool and to illustrate its workings.

## Highlights

• Participatory Design (PD) requires nuanced concepts of accountability and rigour.
• Accountability and rigour are constructed through debate, critique and reflection.
• Our "tool-to-think-with" guides designers in systematic and critical reflection.
• We provide four lenses for reflection: epistemology, values, stakeholders, outcomes.
• The "coherence" between reflective perspectives indicates internal rigour of PD work.

## 1 Introduction

As approaches to designing interactive technology evolve, we continue to see a paradigm shift from the historical engineering mindset, with its focus on requirements, tasks and efficiency, to a holistic, social, situated and human-centred view (Harrison et al., 2011). And with it, a broad consensus in human–computer interaction (HCI) is emerging that recognises that more relevant and meaningful technology can be created by giving people who are affected by it some role in its design. As a result, User-Centred Design and Participatory Design (UCD and PD) approaches have seen significant uptake in recent years. Participatory Design has been re-interpreted and adapted for different design contexts and purposes and we nowadays see a wide spectrum of philosophies driving PD processes, possibly best described as ranging from pragmatic to idealistic (Kensing, 2003). While the historical traits of PD, rooted in the political struggle of labour movements in Scandinavia (Bødker et al., 1987), are more visible on the idealistic end of the spectrum, pragmatic interpretations have focused increasingly on effective design and participation as a means for matching user needs with the affordances of new technologies.

Whatever the flavour of PD, the participation of people in the design process means that researchers, designers and practitioners impart some control over outcomes and processes to their participants. This, in combination with the systematically inherent complexities of contextual dependencies in PD, leads to what is often described as “messy” processes. This makes it difficult to reconcile the practice of PD with traditional science paradigms or epistemological frameworks, which has hampered the field in multiple ways. Firstly, it has made it problematic to communicate the merits of PD to other scientific fields, clients or the public at large. Questions like “*Has participation made a difference and by how much*?” rest uneasily with the nature of the PD approach, as do queries for the “*hard evidence*” for design decisions. Secondly, it has impeded progress within the field of Participatory Design in that the knowledge that is generated is not sufficiently generalisable or accessible to the extent that it can be re-used or built on. Consequently, many wheels are re-invented and much insight lost.

To tackle these issues one might be tempted to “scientise” PD (compare discussion with respect to design in Gaver, 2012). However, PD takes a fundamentally different metaphysical stance, which distinctively sets it apart from the engineering tradition of building interactive technology. Any attempt to retrofit PD with a (post-)positivistic perspective would necessarily make it look scientifically weak, supported by fuzzy data and arbitrary in terms of its conclusions. Instead of seeing the practitioner as an objective observer who inquires about an absolute reality and the best possible solution, PD sees knowledge generation as a dialogic process that is mediated by values and strongly situated. The philosophy that underpins the ideas and concepts of PD are deeply rooted in the postmodern tradition, including phenomenology and Marxism (Ehn, 1989), and demand a different epistemological position as well as methodological approach. So, instead of imposing a positivistic philosophy, we propose that PD needs to build on its own philosophical groundings to argue for its qualities and contributions. The key to constructing these arguments lies with finding a language that reflects the belief system within which PD operates and that enables us to describe the qualities of the diverse work that came to be called PD.

### 1.1 Accountability and rigour

We turn to two inter-related qualities as cornerstones around which we propose to develop such a language: accountability and rigour. By “*accountability*” we mean the ability to link the collaborative work in PD with decisions and outcomes^1^ in a transparent way. The notion of “*rigour*” is commonly associated with a strict positivistic view on science, emphasising universal truths validated by deductive reasoning or measured evidence. In the context of PD we interpret rigour as internal validity, in other words, that a well structured argument can be made for the way a PD process has been conducted. It becomes clear that both terms centre around the quality of PD work, the appropriateness of its methodology and the solidity of its theoretical grounding. Like two sides of a coin, the main difference lies in the intended direction: while accountability emphasises the communication of this quality to others, rigour is mainly concerned with the internal processes relating to decision making and implementation.

Within the positivistic realm, being held accountable and demonstrating rigour are governed by statistics, logic, deduction and proof. The post-modern scientific paradigm on which PD builds, however, does not allow for a similar certainty and there is no quantitative scale or even binary label for the quality of work; too complex are the contextual interdependencies and too important is the role of the researchers, designers or practitioners whose impact is an integrative and desired aspect of the enquiry. Related fields have faced similar challenges and have started to respond in a variety of ways. Fallman and Stolterman (2010) for example, have discussed rigour and relevance in Design Research along the same lines. They too argue for a shift away from the positivistic tradition in assessing rigour in this field and advocate a nuanced notion of rigour that originates from a deep understanding of the particular purpose of design activities. Wolf et al. (2006) introduce the notion of Design Rigour and, delineating it carefully from the traditional notion of scientific rigour, discuss the professional qualities of design praxis that can appropriately describe good design culture. They also make the point that by highlighting the qualities of such design culture, they dispel the notion of design being perceived as the “black art” in HCI—a challenge not unfamiliar to PD. Action Research (AR) is another example from the social sciences which continues to make the argument for alternative notions of rigour for their work (Greenwood and Levin, 2007, p. 55). There are obvious parallels between PD and AR (Foth and Axup, 2006), unsurprisingly given their shared ideological heritage, but it seems that AR׳s epistemological underpinning is even more radically opposed to positivism as it fully embraces relativism and constructionism (see Guba and Lincoln, 1994, for a useful overview of science paradigms).

From the above discussions, it becomes apparent that accountability and rigour in a post-modern scientific context is delivered through debate, critique and reflection. For example, Wolf et al. (2006) highlight the ‘design crit’ as one of the qualities of design practice that contributes to its rigour. They define it as “*... a designer*׳*s reflective, evaluative and communicative explanation of her design judgments and the activities in which she has engaged*.” However, for PD to take part in such a debate about rigour and accountability, we must develop a language that allows us to communicate such an explanation and to construct solid arguments for the quality of the work. Since many of the features of PD are tacitly embedded in its practice, critical reflection is the key to becoming aware of its qualities and thus to developing a language for arguing rigour and accountability. It is here that this article aims to make its main contribution: we propose a conceptual framework to support designers, researchers and practitioners conducting Participatory Design work to engage in a process of critical reflection and, as such, give them the language needed to convey the rigour and accountability of their work.

### 1.2 A tool for whom to do what?

The conceptual framework we propose is a “tool-to-think-with” that we argue should become an integral part of a reflective practice in Participatory Design. It guides designers, researchers and practitioners in incorporating phases of critical reflection with the goal of giving them the means to reify the rigour inherent in their practice. The awareness and the language this guidance affords, also offers appropriate means to explain decisions and judgements to the outside world and thus allows designers to increase their accountability.

We argue that such a “tool-to-think-with” can benefit PD practice at all stages. Firstly, when planning and setting up PD work, underlying assumptions and tacit forces can be brought to the fore, allowing, designers, researchers and practitioners to make more considered decisions on methodology and organising involvement. Secondly, during the design work proper, the tool supports designers in responding to new situations and in steering the process, guided by an increased awareness of what are the drivers. It also aids in explaining PD to involved stakeholders in this phase, be they participants, clients or co-researchers. And thirdly, once the project is finished, it allows designers to critically reflect on their work and describe the knowledge, the contributions and the lessons learnt, which is crucial in allowing PD to evolve as a field. This tool aims to provide a language that enables us to have a debate about what works when and why. As such, work can be scrutinised more effectively and transparently, and avoids PD being judged against positivistic standards it was not designed to meet.

Our “tool-to-think-with” consists of four lenses, *epistemology*, *values*, *stakeholders* and *outcomes*. These lenses guide the inquirer in taking different perspectives to critically reflect on their work and thereby discover qualities that otherwise might remain tacit. Furthermore, we examine the *coherence* between those lenses, i.e., the extent to which the fundamental qualities of a PD effort are attuned to each other. We argue that this *coherence* is a prime indicator of rigour in PD work and a powerful concept that reveals how coherent a process was, how appropriate the methodology was and how well informed decisions were. As we will further discuss below, the concept of *coherence* does not mean to imply *agreement* within the perspectives, i.e., we are not suggesting that, for example, values need to be agreed upon for a work to increase its rigour. Analysing the *coherence* makes no judgements about where on the broad spectrum of PD the work is situated, indeed, it aids practitioners in positioning themselves on this spectrum. It encourages practitioners to characterise their tradeoffs and standpoints, and argue for rigour within their chosen approach or philosophy. This framework is not intended to be a cookbook with a set number of metrics that result in a measurement of rigour, as PD efforts are too contextualised and varied for this to be meaningful. Instead, it provides an empowering basis from which designers, researchers and practitioners can build strong arguments for the value of their work.

### 1.3 Outline

The article is structured as follows: to ground these arguments, we first reflect on the participatory design work conducted in a multidisciplinary, distributed project called ECHOES, where PD was “owned” by one strand of the project, and had to negotiate its contributions alongside other concerns. The experiences described here provide a concrete scenario which served as a starting point for motivating the development of the framework. Rather than reporting on the methods and outcomes of the PD work within ECHOES, which have been published elsewhere (Frauenberger et al., 2013, Frauenberger et al., 2012a, Frauenberger et al., 2012b, Frauenberger et al., 2011, Porayska-Pomsta et al., 2011), we focus on the challenges and opportunities inherent in the process and, as such, provide a chronological account of the PD work as it unfolded. The *beginnings*, *middles* and *ends* sections tell the story of a struggle to implement a PD process given the many contextual constraints, ideological misunderstandings and practical necessities. The themes of this struggle allowed us to develop the framework for reflection, which we introduce in a subsequent section. We then discuss the concept of *coherence*, its relevance as an indicator for rigour and accountability, and the practical implications of our concepts. We close by summarising our contribution and laying out our plans for future work with this framework.

## 2 The ECHOES project

We use the ECHOES project as a case here, because it illustrates challenges and tensions in trying to contribute a PD stance in a project that had many other constraints and requirements, and involved partners from various different scientific cultures. Two of the authors (Good, Frauenberger) were directly involved in ECHOES, their main responsibilities being to plan and conduct PD activities to support the overall development of the system. Although we describe a particular experience from a particular perspective here, we believe that many of these challenges and tensions are typical for PD work, if not in the exact same configuration. We also realise that other PD projects may have experienced more internal agreement, but ECHOES provides valuable insights as a case study, because the PD strand was regularly challenged by the other project members about its position on the PD spectrum, the validity of its outcomes and rigour of its work.

**Fig. 1:**
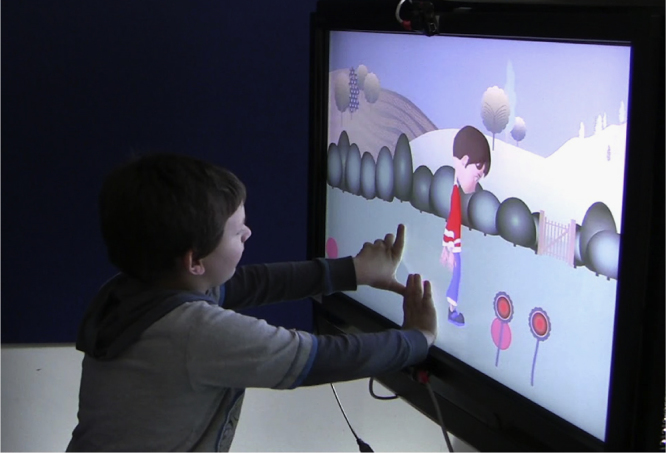
A child playing with the finished ECHOES system.

The project set out to develop a technologically enhanced learning (TEL) environment for typically developing children and children with autism spectrum conditions (ASCs). The goal was to scaffold the development of children׳s social skills through a series of playful learning activities that take advantage of virtual characters, multi-touch surfaces and advanced sensing technologies. We thereby sought to exploit the natural affinity that children have with computers, particularly those on the autistic spectrum (Murray and Lesser, 1999), and provide a motivating environment (Porayska-Pomsta et al., 2011). The project׳s target population was typically developing children between 5 and 7 years of age and children on the high-functioning end of ASC of an equivalent developmental, if not chronological, age. ASCs are characterised by a triad of impairments related to social skills, communication and rigidity of thought. Children with high-functioning ASCs tend to exhibit relatively typical pragmatic language and cognitive abilities, but do show impaired skills in social communication and a tendency towards narrow interests. Fig. 1 shows the finished system in action.

ECHOES was funded under the Technology Enhanced Learning (TEL) programme,^2^ a joint initiative of the EPSRC (Engineering and Physical Sciences Research Council) and the ESRC (Economic and Social Research Council), two national funding bodies in the UK. This meant that the educational aspect in the research was seen as central. The composition of the project and its prior planning demonstrate that PD was not an intrinsic position, but one of a number of aspects to aid the creation of the system. As such ECHOES was not a fully committed “PD project” per se, but had a PD component in which researchers had to involve other members of the team as well as children, parents and teachers as stakeholders with particular interests. Consequently, while the issues might have taken a particular form, we believe that the underlying challenges are indicative for many PD situations.

The following describes the PD work in ECHOES from a historical perspective: “The beginnings” looks at the initiation of participation, the planning and our expectations. “The middles” section is concerned with how the work was implemented, how the researchers responded to new challenges and fluidly adapted the process. Finally, “The endings” section discusses how the work was wrapped up and what remained when we left.

### 2.1 The beginnings

The initial workplan in ECHOES was organised into four strands: (1) Learning Activities, (2) Participatory Design, (3) Technology and (4) Evaluation. Each strand consisted of one strand leader and between 2 and 4 associated researchers. The Learning Activities (LA) strand was responsible for developing activities for children that were grounded in SCERTS (Prizant et al., 2005), the psychological intervention framework used in ECHOES. SCERTS allowed the researchers to define measurable learning objectives and develop a planning engine that would intelligently drive these activities and their sequencing. The planning was to employ Artificial Intelligence (AI) techniques to reason about the system׳s behaviour based on the child׳s input and on additional data collected from a vision system that deduces the emotional state of the child in front of the screen through facial recognition algorithms. The Participatory Design (PD) strand, of which one author was the leader and another an associated researcher, was intended to implement a learner centred, participatory design process to involve children, carers and teachers throughout the project to develop the system. Strand 3, Technology, focused on the technical implementation of the system and the integration of the multiple parts (planning engine, rendering, multi-touch input, vision, etc.). The first author of this article was also associated with this strand and collaborated with others on developing and implementing the system. Finally, the main task of the Evaluation strand was to conduct a comparative intervention study that aimed to investigate the effects of the ECHOES system on children׳s social skill development.

In the research proposal, the goals of the PD strand were defined somewhat ambiguously and left room interpretation for where on the PD spectrum the work would be. While it stated that PD would seek to involve children, teachers and carers as design partners to “*support the co-evolution of ECHOES*׳ *II learning activities and tools ... from the outset*” it defined the scope of the related workpackage as focusing on the system׳s interface. This ambiguity and its implicit divergence in expectations may have marked the start of the PD strand׳s struggle to find its role within ECHOEs and to develop a well defined working relationship with the other strands. Uniquely, the project planned to use two groups of children in parallel: typically developing children and children with ASC of corresponding developmental age. Methodologically, the PD strand aimed to build on the CARSS (Context, Activities, Roles, Stakeholders, Skills) framework (Good and Robertson, 2006) and develop participatory activities with learning in mind. The involvement of domain experts, teachers and carers was to be achieved primarily through focus groups and specific workshops.

In operationalising the goals from the research proposal, the PD strand faced a number of initial challenges. The team set out to frame the design problem from various angles and to develop a process that would lead to appropriate participatory activities that could inform the design. Importantly, the researchers needed to consider project needs on the one hand, and the kinds of experiences children should have in the process on the other, before going on to determine whether these were in conflict. A major practical issue at this point was that all strands began work at the same time. While learning activities and technologies were already being developed, and input was expected from PD, the PD strand had to carefully set up collaborations with schools and recruit participants. Participatory work with children with disabilities requires a long lead time, and the careful development of relationships based on trust. Key challenges were finding partner schools that were willing to collaborate, implementing a systematic ethics procedure and fulfilling the formal ethics requirements at each partner university as well as UK National Health Service (NHS) ethics, winning over parents and teachers, producing the necessary informational materials to support these efforts and, most importantly, building up a strong relationship with the children. It took several months before the team was able to conduct the first workshop in a school, however decisions around technology use and learning activities were already being made or assumed elsewhere in the project.

In these beginnings, expectations were formed in all strands with respect to potential outcomes and the ways in which they should be achieved. While not yet fully evident, these expectations were quite diverse, rooted in the different backgrounds of the individuals, and the scientific cultures in which they were embedded. In terms of process, the PD strand strongly embraced the ideological notion of empowering children, trying to carve out as much scope for impact as possible. With respect to a product, the team envisaged the co-creation of an open ended, exploratory and playful environment, in which learning would occur naturally and implicitly. Conversely, the LA strand had strong views on the learning elements, intrinsically motivated by psychology, and expected PD to focus on developing specific activities that children would like. The Technology strand had clear expectations in terms of the potential of the technology available to them. In this phase, the vision system in particular seemed to provide an attractive technological challenge that would play a key role in informing the planning of activities. Finally, the Evaluation strand expected a system that would allow impact to be measured. Negotiating these divergent expectations was complicated by the physical distribution of the team and while there were regular project meetings, open as well as tacit differences persisted throughout the project.

### 2.2 The middles

Once the practical pre-conditions for the participatory work had been established, the first PD activities were developed and implemented. Meanwhile, the project team continued to struggle to come to an agreement over the best working model, particularly with respect to the relationship between PD and the other strands. PD was widely seen as an information provider, the part of the project in which the look-and-feel of the system would be determined by studies that elicited the children׳s preferences. As a result, a question-and-answer working model was favoured where particular questions like “Which objects will children engage with on a screen?” would be put to the PD strand which would elicit specific answers from participatory studies with children. The PD strand resisted this notion and argued against PD being seen purely as a requirements elicitation effort, instead advocating a more holistic approach that allowed for true co-creation. Several features of PD were in particular conflict with the research cultures that dominated other strands: firstly, that it is explorative and that it is neither desirable nor possible to know the range of solutions that PD work would create. Secondly, that results are not quantifiable in the usual scientific sense (e.g., a preference rating of objects). Outcomes may require interpretation, are less specific and form only part of the design solution. And thirdly, the knowledge constructed in the collaboration with participants does not necessarily follow from the questions one asks, but leverages an empathetic understanding of participants and their actions.

The PD strand initially conducted a series of sensory explorations with children to design a plausible and meaningful environment in which the learning activities might take place (Frauenberger et al., 2011). These explorations yielded a wealth of rich input from children as well as a greatly increased understanding of the physical, social and health related contexts of the design. Much to the frustration of the other strands, however, the PD strand could not provide straightforward answers to the questions posed. The question-and-answer clearly showed its limitations as the PD strand too was frustrated because of the lack of understanding for the kind of insights PD provided. There was a clear sense that PD would have needed to be much more deeply interwoven and embedded within the work of other strands. Translating the input into a design posed a major challenge for the PD team, not least because design decisions needed to be justifiable and supported by the “data”. In response, the PD strand sought to develop a systematic and transparent process to bridge this gap (Frauenberger et al., 2010) and in an internal workshop, PD work from over a year was used to develop a design for the system that was true to children׳s input and allowed the required learning activities to take place (Frauenberger et al., 2012b). The key components in this process were a mindful interpretation of children׳s contributions that considered the input as much as the empathetic understanding of the designers, a phenomenological analysis that looked beyond literal meanings to gain an understanding of desired experiences and the notion of input as design triggers. The PD strand communicated the design results, including the rationale, through video walkthroughs, storyboards and case studies with child personas.

With the overall design language decided, the environment needed to be populated and learning activities embedded within it. In a second series of PD activities, the PD strand explored the notion of a magic garden with children to take explicit advantage of the digital domain as a highly flexible and imaginative world (Frauenberger et al., 2011, Frauenberger et al., 2012b). Again, through a process of mindful interpretation of the children׳s ideas we arrived at a number of design concepts within the garden environment that were able to support the learning activities. These included, for example, flowers which children could grow in pots and transform into bubbles by flicking their heads. The prospect of being able to pop a bubble was motivation for many children to follow a social interaction with the virtual character in the scene about growing the flower.

Reflecting on this phase of the project, the way in which PD integrated with the project steadily improved as design decisions were being made. However, in the process the PD team felt it was lacking an appropriate language to communicate its work effectively to other strands. Disagreements and misunderstandings about the kind of work PD tried to do could not be resolved, because the different research cultures made it difficult to find common ground. Within the PD strand itself, a number of other fundamental questions also began to emerge: how much of an impact are the children really having on the design? Are they in any way empowered by their participation? Is the PD team not merely a proxy and if so, which implicit assumptions within the PD team are being portrayed as children׳s input?

### 2.3 The ends

Nearing the end of the project, there was increasing pressure to deliver on the central scientific outcome promised in the research proposal: a controlled, large scale intervention study investigating the impact of the system on the development of a set of social skills in typically developing children and children with ASC. In this phase, the project faced two main challenges: firstly, to complete the design and implementation of a stable system that could be taken into schools and be used to collect the necessary data; and secondly, developing a study design that controlled for the widely varying contexts and populations in which the system was deployed and enable data to be collected that could be analysed in meaningful ways.

The PD strand was minimally involved in this evaluation effort, and since there was no scope for altering the design at this point, focused instead on an alternative approach to evaluating the system and developing starting points for future work. To this end the PD team developed a PD Critique activity that aimed to conduct a series of design critique sessions with children with autism (Frauenberger et al., 2013, Frauenberger et al., 2012a). While the initial goal of the activity was quite specific, the outcomes exceeded the expectations and revealed far more substantial insights than the team had originally envisaged. Crucially, the activity demonstrated the potential role of simple, digital tools, to support complex interactional needs of children with autism and allow them to successfully navigate difficult social situations such as a design critique. While these outcomes had no immediate bearing on the ECHOES system, they furthered our understanding of how to meaningfully and sensitively engage children with ASC in the design process, and highlighted a number of promising avenues for future research.

Starting to look back, the PD team was also in search of its legacy. While there was a strong sense that participants had enjoyed the collaboration over the course of the project, questions remained over whether the collaboration could be considered successful, in what ways, and for whom. The PD team reflected on the quality of the participation, on whether there had been sufficient scope for impact and the degree to which opportunities for handing over control to participants had been taken. Also, the PD team had been careful not to end collaborations abruptly, instead organising farewell sessions with participating children, presenting them with tokens of appreciation for their involvement (certificates, badges, etc.), creating films of the participatory work we conducted which were screened at school assemblies and given to participants and their families. However, the question remained as to the extent to which children benefited beyond these little rewards, and in what ways, or whether it was enough for us to benefit in terms of a “better” design and for them to have a good time.

## 3 A conceptual framework for reflection

The experience in ECHOES motivated us to develop a “tool-to-think-with” that would allow PD practitioners and researchers to assess the level of rigour and accountability in their work and effectively communicate it to others. The challenges and tensions emerging from ECHOES provided us with starting points: to analyse PD work, a notion of internal rigour has to consider the interplay of stakeholders, researchers and participants on multiple levels. ECHOES has demonstrated the impact of divergent motivations, scientific cultures and value systems. It has highlighted how tacit differences in expectations and projected outcomes hampered the development of a consistent methodology, and how competing priorities and external requirements curbed the scope of PD. Complex social interactions between stakeholders played a key role in making some aspects succeed and others fail. Simple practicalities such as different timeframes for work or access to participants were also key factors. Furthermore, ECHOES showed that benefits and gains are difficult to assess, because of the multitude of perspectives one can take on this question.

We argue that the quality and coherence of solutions that PD work finds in dealing with these challenges and tensions can be seen as prime indicator for its internal rigour. To unearth these mostly tacit aspects in PD work, a process of critical reflection is needed. Here we build on the work of Schön (1983), Sengers et al. (2005) or Bødker and Iversen (2002) who all argued for the necessity of a reflective design practice when faced with “wicked” problems (Rittel and Webber, 1973), problems which defy ordinary problem-solving techniques, problems like those in PD.

**Fig. 2:**
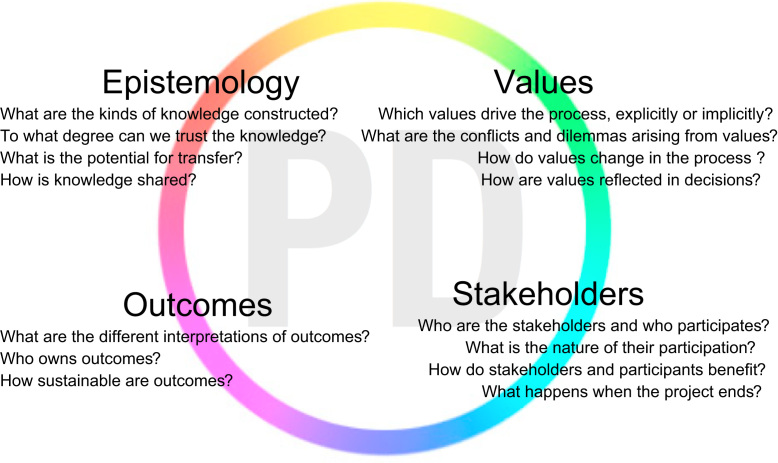
Summary of the four lenses and starter questions in the conceptual framework.

Thus, our “tool-to-think-with” guides practitioners and researchers in a process of structured, critical inquiry into the tacit qualities of their PD work. It is composed of *four lenses*: *Epistemology*, *Values*, *Stakeholders*, *Outcomes* which each pose a number of starter questions to point the practitioner to different directions of critical inquiry into their work. Fig. 2 provides an overview before we discuss each lens and their starter questions in more detail below. We are aware that there are other possible ways in which reflection on PD work could be structured, but we believe these four lenses to be an appropriate starting point.

### 3.1 Epistemology

When Participatory Design is conducted in a scientific research context, as is the case for much of the work referred to in this article, the contribution to knowledge is an inherent and dominant driving force. However, we argue that even when research is not the explicit goal, the nature of PD implies gaining understanding and a generative creativity that in itself leads to different ways of knowing. Thus, knowledge construction is an integral part of conducting PD, underpinning its processes and outcomes.

While the term “knowledge” and the language around it is typically associated with the positivistic science paradigm, PD׳s relationship with the positivistic perspective on knowledge generation has not been an easy one. In contrast to results in the traditional sciences, PD outcomes do not typically lend themselves to being quantified, compared, generalised or replicated. The epistemology is inherently co-constructed, situated and embodied, like in many other designing disciplines. Thus, when we use “knowledge” here in relation to the kind of insights PD produces, we also implicitly argue for acknowledging different ways of knowing. The field of HCI has been at the forefront of this argument and there has been a growing recognition of the need for situated approaches to be able to describe and understand the shifting application domains of technology. Harrison et al. (2011), for example, argue for a paradigm shift towards the “3rd-wave” HCI as a successor science that is based on situated meaning making, a standpoint epistemology, values and alternative evaluation approaches. And more recently, Olson and Kellogg (2014) have collected a number of different perspectives in their book “Different Ways of Knowing in HCI”. PD׳s basic qualities align themselves well with this emerging paradigm, explaining to some extent the rise in popularity of participatory and human-centred approaches in HCI.

There are two major strands of research that provide a valuable background to framing the epistemology of PD. One is Design Research, or Research through Design, which investigates how knowledge and theory emerge from applying design practice as a method of inquiry in HCI (Zimmerman et al., 2010). The other is PD׳s sister field in the social sciences, Action Research (AR), which aims to create a new understanding of people׳s practices by becoming part of the practice and to bring about change by action that is informed and shaped by this collaborative understanding (Greenwood and Levin, 2007).

AR shares many of the underlying values and goals with PD, such as empowerment an democratisation, and consequently also some of its methodology. As Foth and Axup (2006) find, the main differences lie in the intent and purpose: while AR might be characterised as seeking to act, change, understand and reflect, PD is more concerned with involving and designing. AR׳s more mature state as a field means that the epistemological argument is similarly more well developed and, unlike PD, AR makes the construction of knowledge a primary goal of the process. However, both PD and AR work is highly contextualised and arguing for scientific rigour leads to the same epistemological difficulties. AR׳s starting point is to reject positivism, with its notions of abstract knowledge in an absolute world, in favour of Lewin׳s pragmatism and a hermeneutic philosophy in which the world is available only subjectively, and constructing knowledge means negotiating interpretations of this subjective world (Greenwood and Levin, 2007, p. 56). Thus, knowledge in AR is co-created and context bound. Its rigour stems not from validity and reliability (compare Fallman and Stolterman, 2010), but from trustworthiness which we will discuss in more detail below (Guba, 1981, Greenwood and Levin, 2007). Hayes (2011) provide a more complete discussion about the value of AR for HCI, concluding that “*AR offers HCI researchers theoretical lenses, methodological approaches, and pragmatic guidance for constructing credible knowledge alongside collaborative projects...*”.

Design Research has also found itself debating its epistemological foundation due to its poor fit with the prevailing positivistic stance in traditional sciences (Fallman and Stolterman, 2010). At its core the issue seems strikingly similar to that of AR: how could localised and highly contextual, creative design practices yield scientific knowledge of appropriate rigour? Cross (2001) first discussed the “*different ways of knowing*” in design practice and identified the following main kinds of knowledge in design: knowledge through the activity of designing and reflecting, knowledge inherent in the artefacts and the process, and knowledge through the teaching of others. There seem to be two main views, however, on how these kinds of knowledge should be constructed: one calls for a systematic and disciplined approach to demystify the “black art” of design, while the other argues that such a move would run counter to the essence of Design Research. In the first instance, Zimmerman et al. (2010), for example, argue for a unifying methodology with guidelines and protocols, more research examples with knowledge creation in mind and a critical reflection on theory. Similarly, Zimmerman et al. (2007) propose a framework of four lenses to evaluate contributions to knowledge systematically: transparency and rigour of the design process, the level and quality of invention, the real-world relevance and the re-usability of outcomes. However, it remains open who would be best qualified to judge aspects of invention or relevance. On the other hand, Gaver (2012) questions this push towards conformity and standards in research through design as he sees the diversity and non-convergence as the defining asset design has to offer to HCI.

Against this background, we have developed the following starter questions for our framework:

*What kinds of knowledge are constructed*? We identify four broad and often overlapping types of knowing that can emerge from PD work: social, design, methodological and theoretical knowledge. By social knowledge we mean the local knowledge gained from a PD process about the social environment in which it is embedded, and its unique dynamics. Grönvall and Kyng (2012) provide a typical example when describing a participatory approach to designing home-based healthcare. The process not only produced concepts for technology, but a much greater understanding of the life-worlds of the elderly in their homes. Design knowledge refers to the knowledge embedded in the actual artefact or service—the design. As Cross (2001) points out, the design artefact embodies decisions and considerations and thereby is a manifestation of knowledge. By affording experiences, designs also embody knowledge about the practice of people interacting with them, which can overlap with the social kind of knowledge described above. Methodological knowledge is constructed by the application, adaptation or innovation of methods in PD. As Zimmerman et al. (2007) highlight, rigour and transparency in the process is a major contribution to knowledge in Design Research and a majority of PD research papers indeed focuses on techniques and methods (see also Kyng, 2010). Finally, theoretical knowledge relates to attempts to construct theories from PD processes. In line with the epistemological stance PD takes, theories are unlikely to possess the predictive power of theories in the positivistic realm, but are generative and aspirational. They are also provisional and contingent, but as Gaver (2012) points out, this is desirable in that they have the power to point to new realities.

*To what degree can we trust the knowledge*? We have argued above that the kinds of knowledge PD processes construct do not sit well with a strictly positivistic epistemology. Action Research and Design Research, however, provide conceptual frameworks that allow for an alternative assessment of the quality and rigour of knowledge in their respective fields. A central concept to replace positivistic validity or generalisability is *trustworthiness* which stems from four distinct properties: credibility, transferability, dependability and confirmability (Guba, 1981). Credibility involves two distinct perspectives: firstly, internal credibility established by participants and their assessment and acceptance of outcomes—the practice test. Secondly, external credibility requiring external judgements about how believable outcomes are given the supporting evidence from the process (Greenwood and Levin, 2007, p. 66). Dependability and transferability will be discussed below in more detail and confirmability is the extent to which the process can be repeated by others without changing fundamental insights. Shenton (2004) provides valuable strategies for reinforcing each of these aspects so as to increase trustworthiness in qualitative research. Two of the lenses described in Zimmerman et al. (2007) are also relevant here: one is the level of innovation, while the other is the level of relevance in the real world. However, in contrast to *credibility*, which specifically defines internal and external perspectives, innovation and relevance are portrayed as objective measures in Zimmerman et al. (2007) which PD would argue can vary with the perspective taken.

*What is the potential for transfer*? Rejecting generalisability does not mean that knowledge constructed in PD processes can have no relevance in contexts other than the ones in which it was created. Following the epistemology of Design Research and Action Research, the alternative which has been argued for is contextual dependency and transfer. As Guba (1981) discusses, dependency is the direct counterpart of the positivistic concept of reliability, referring to the absoluteness behind reliable measurements. Dependability, in contrast, seeks to account for different realities by linking knowledge to context. Understanding how knowledge *depends* on the context is a pre-requisite of being able to *transfer* it to other contexts. Stolterman (2008) makes a similar argument when contrasting the ways in which design practice and positivistic science deal with complexity: design is situated in a real-world complexity that is not deducible to definite solutions, and design briefs are inherently wicked problems (Rittel and Webber, 1973). As such, design knowledge can only be understood within its context.

*How is knowledge shared*? We expect that many designers or researchers reflecting on projects through these questions will find that much knowledge and insight remains tacit or unarticulated. While tacit knowledge can also be transferable in certain situations, e.g., in an apprentice model, there are many other situations where knowledge transfer benefits from knowledge being formulated and made explicit. Not least, academic communities and their meetings and conferences are venues where explicit knowledge, as in the publications of a conference, are used to further the state-of-the-art and help researchers to build on the work of others. Knowledge can assume many formats ranging from theories to methodologies, frameworks, guidelines, tools, case studies, design patterns or policies. Each format addresses the needs of a specific audience and implies appropriate ways of publication. The question about availability indirectly relates to ownership as well, in the same way as outcomes are typically owned.

### 3.2 Values

Values, in the broad sense we are using the term in this article, are ideas or qualities that individuals or a group of people consider to be of importance and worth in life^3^Friedman et al. (2008) note that values cannot be motivated by facts of the external world, but depend primarily on “*interests and desires of human beings in their cultural milieu*”, highlighting the fact that values are as multi-faceted as human beings themselves, and cannot be proven, disputed or declared invalid per se. They are subjective to the individual or the group, and collaboration or even simple interactions require some form of negotiation of values.

The significance of values in designing technology has been recognised for some time as an underlying driving force for the aesthetic, practical and moral judgements of human beings (e.g., Friedman, 1996). HCI in particular has opened up to the concept of values and aims to build on sociology and anthropology to understand the role of values in how we interact with technologies. Sellen et al. (2009), for example, argue that with the prevalence of technology in our future digital lives, it has become indispensable to find ways to design for diverse human interests and aspirations, and they make the case for “*folding human values into the research and design cycle*”. However, in addition to the need to design for user values, as Sengers et al. (2005) point out, designers and researchers also bring their own values to the design process. As such, careful reflection on practices and decisions is required in order to understand the impact of these values on a design process.

Participatory Design׳s historical context explains why it is inherently concerned about values. From its beginnings, values such as *democracy*, *empowerment* and *empathy* were deeply engrained in the methods of PD, in fact they were the main reason for PD׳s existence (Bødker et al., 1988, Ehn, 1989). Thus, in contrast to many other academic fields or practices, PD not only recognises the significance of values, it also *stands for* values that go beyond or even against the traditional values of science such as *universal objectivity*. This is true to a lesser extent for the more pragmatic interpretations of PD, but it is important to recognise that choosing a participatory approach in itself is an expression of values that designers or researchers bring to a project.

While much of the recent PD work is concerned with methods and techniques (Kyng, 2010), Iversen et al. (2010) argue for a value-led participatory design approach. They see a co-design process, at its core, as a negotiation of values that all participants bring to the table or which emerge from the collaborative experience. Consequently, they see their own task as establishing a culture of dialogue and discourse through which they “*cultivate the emergence of values, develop the values and ground the values*” that inform the design (Iversen et al., 2012). Values are collaboratively developed, questioned and re-conceptualised possibly giving rise to conflicts and dilemmas. PD activities should be specifically designed to allow existing values to surface and new ones to emerge. Finally, values are grounded in everyday practices of stakeholders and realised in design artefacts which embody the refined and negotiated values.

Halloran et al. (2009) arrive at a similar conceptualisation of the role of values in their discussion of resourcing the design of ubiquitous computing through values. Importantly, they too highlight that values are unlike given requirements, but change in response to the co-designing process. They add that values have the potential to mediate between stakeholders and support the engagement of participants.

For the framework, we derive the following starter questions from these discussions:

*Which values drive the process, explicitly or implicitly*? Awareness of values is the necessary precondition of evaluating the ways in which they shape the design process. However, values are not abstract entities, but originate from participants, researchers, designers or organisations. Explicit awareness of all values involved in a project is rarely achievable as participants do not usually state their values openly unless co-design activities are specifically designed to elicit them. More commonly, values are expressed implicitly in the way we interact, for example by how we engage with aspects of the design process, what and how we contribute and envision or what we agree or disagree with. Values are also embedded in all decisions made before co-design activities commence – e.g. in the design brief, the goals and the chosen methodology, but also through the frameworks of funding bodies and the scientific cultures in which projects operate. Furthermore, designer and researcher values are likely to be implicit in the types of activities which form the basis for participatory design sessions. In addition, the visibility of values amongst participants is also important, because it is a key component of understanding co-participants and engendering an empathetic discourse. So, this question is not only about which values, but also whose values drive the design process and how much this is visible.

*What are the conflicts and dilemmas arising from values*? Many conflicts in co-design can be best understood by knowing what motivates different interests, and understanding the value system involved is likely to be a key piece of the puzzle. Conflicts and dilemmas, however, are not necessarily undesirable, in fact, like other design oriented approaches, PD views them as a resource and an opening for invention (Gregory, 2003). For example, Iversen et al. (2010) report that their iSchool project led to a dilemma regarding the roles of teachers in relation to the new technologies envisioned. The conflict, rooted in the traditional values of teachers, offered an opportunity to re-conceptualise and re-frame the problem. Guided by PD activities, teachers began to imagine novel roles for themselves in which they were empowered by technology, rather than threatened by it. As Gregory (2003) points out, embracing dilemmas and contradictions in this way provides “*openings for expansive transitions that go beyond situated problem-solving*”. Thus, recognising value-based conflicts also means recognising potential for change and invention.

*How do values change in the process*? A consequence of collaboration in design is the negotiation of values, whether they were explicitly articulated or implicitly present at the start, or emerging from the process. Both Iversen et al. (2010) and Halloran et al. (2009) highlight the fact that values change in response to participation and this question asks which of the values kept their significance throughout the process and which have changed and why. The Chawton House project, for example, aimed to explore the use of mobile technology in guiding visitors around an old English country estate. The curators who participated in the co-design experienced a shift in the values engrained in their practices as they discovered the possibilities of technologies. Equally, the researchers came to appreciate the value of the authentic enthusiasm and narration skills of the curators and their voices were used directly in the resulting system (Halloran et al., 2009). This example speaks to the mutual learning aspect of PD, often stated as an intrinsic motivation. The authors also observed that values become connected to activities and artefacts, so in evaluating the evolution of values within a PD process it is important to determine the role that activities and artefacts played in mediating the negotiation of values.

*How are values reflected in design decisions*? Values are not only linked to activities and artefacts, but are inherently connected with the design decisions that preceded them. As Bratteteig and Wagner (2012) show, they are not necessarily aligned with the outcomes of the collective negotiation process, and many decisions are made implicitly, are influenced by external forces or are a consequence of unequal power structures. However, identifying the values reflected in design decisions is probably the single most effective way of assessing the role of values in a PD process. Even when decisions are not made collectively, for example, because the participant group cannot be expected to contribute at this level, we believe tracing values from participant input to design decision is still important. Frauenberger et al. (2012b), for example, described several workshop activities with the specific aim of interpreting input from children with ASC, starting by collecting values as *must-haves* for their design. Similarly, Iversen et al. (2010) discuss the translation of values towards design ideas. Their concept of an *appreciative judgment of values* through which designers facilitate the emergence, development and grounding of values highlights the empathetic and reflective role of designers and the ways in which they shape the design decisions by their own values and choices.

### 3.3 Stakeholders

At its beginnings, PD focused on labour contexts, making workers and management, or employees and employers, the natural primary stakeholders in PD projects (Bjerknes et al., 1987). Adding the researchers in their mediating and facilitating role, a typical PD process involved three fundamental camps with relatively clear motivations and goals. With the diversification of contexts to which PD became applied, the range of stakeholders has equally become more diverse, bringing different motivations, goals and values to a PD process. Indeed, in many cases merely identifying all of the stakeholders impacted by the design became less than obvious. Additionally, many PD projects involve stakeholder groups which are too large to be meaningfully involved in their entirety, which requires careful choices in terms of representation and means of participation.

The proceedings of the 2012 conference on Participatory Design exemplify the wide spectrum of stakeholders who are involved in design projects. These range from government organisations and the general public at large (Gidlund, 2012) to specific user groups such as people with aphasia (Galliers et al., 2012) or communities confronted with disaster response planning (Light and Akama, 2012). While not always reported on explicitly, the contexts in which these PD projects are conducted point to complex stakeholder profiles with highly intricate relationships. Gärtner and Wagner (1996) have proposed actor-network theory as a tool to analyse the structural relationships of participant groups in PD projects. In this social theory, human participants are not the only actors in a network: artefacts, concepts and the design itself function as intermediaries. The evolution of the network over time describes the design process and reflects the dynamic relationship between actors. Inspecting PD processes through actor-network analysis, Gärtner and Wagner (1996) identified three main social arenas for PD work: designing work and systems, designing organisational frameworks for action and designing the industrial relations context, in other words, PD within the project, within the organisation or within the broader context of policy and public debate. Thinking of stakeholders in PD as a network of actors in these arenas is useful as it allows the reflective researcher or designer to understand cultural practices, power relationships and the roles of mediating artefacts or concepts.

Design is decision making, and decision making “is the exercising of power” (Bratteteig and Wagner, 2012). Designers and stakeholders negotiate decisions and the outcome relies on the underlying power structure which defines how much scope for change each participant has. Bratteteig and Wagner (2012) have used this perspective as a starting point for a thorough analysis of a long-term PD effort in order to reveal the kinds of decisions that are being made and the mechanisms through which power relationships between stakeholders lead to these decisions. They found that important decisions often fall outside of the temporal or organisational frame of a project and are adopted as uncontested facts or given pre-conditions. While power sharing is at the heart of PD, they also found that many decisions were based on various stakeholders exercising their power implicitly through their expertise, skills or organisational standing in relation to others. Power related concepts such as loyalty, trust or influence played significant roles in how decisions were made. It is therefore key for this evaluative perspective to gain an understanding of which power structures are motivating decision making, both within a project in terms of the participants and in the wider context of the project, e.g., its embedded academic culture or funding structure. Light (2010) also reminds us that interaction, and thus participation, is shaped on multiple levels and that the micro-level, the level of group-dynamics and individuals, is receiving too little attention in terms of how it impacts on exercising power or making decisions.

The starting questions for our framework are as follows:

*Who are the stakeholders and who participates*? As argued above, this question is not as obvious as it seems and does not stop with merely identifying peers affected by the design. Instead, we argue that understanding stakeholders is a multi-layered process, which in turn contributes to understanding the dynamics of collaboration in co-design. Firstly, we believe it is important to reflect on stakeholder motivations and interests in order to gain an empathetic understanding for their actions. This involves looking beyond the immediate groups of people involved, recognising the cultural and professional practices in which stakeholders are embedded in. For example, Bratteteig and Wagner (2012) describe how architects participating in their project felt a strong need for peer recognition in their own field and therefore were in conflict over the imperfections of the novel solutions proposed. Cultural practices can subtly influence stakeholders, for example in academia with a strong “publish or perish” culture, researchers are pushed towards the types of studies which yield publishable results, and thus the culture shapes their role in the collaboration. A related aspect is the issue of representation. An understanding of how certain stakeholders came to be participants, for example by selection, recruitment or driven by self-interest, contributes to the understanding of their actions during the process. Secondly, the relationships between the stakeholders have significant impact on decisions made and therefore on the design outcomes. Recognising power relationships and the mechanisms through which this power is exercised is vital. Again, Bratteteig and Wagner (2012) provide an example where lay participants did not have the technical expertise for making certain design decisions and it required trust to overcome an unequal power relationship with more knowledgable design team members.

*What is the nature of their participation*? In addition to the “who”, the “how” and “to what extent” are equally important. In their review of early PD projects, Clement and Besselaar (1993) have identified 5 key ingredients for participation: participants must have access to information, they must have the possibility for taking independent positions on the problems, they must be involved in the decision making in some way, appropriate methods for participation must be available and there must be the scope for change. However, each of these ingredients may be present to various degrees and thereby shape the nature and quality of the participation. Druin (2002), for example, has identified four levels of participation for children in PD work: users, testers, informants and design partners. Each of these roles assumes influence on the process to a different extent, and while a useful overall categorisation, it does not always capture the nuances of many design situations. Firstly, participant roles tend to be fluid and change over the course of the project (compare Frauenberger et al., 2013). And secondly, it is easy to see how, in theory, full design partners might be in a position to make any decision they like, but are not provided with enough information to do so. Similarly, elaborate methods for participation may be in place without there being sufficient scope for change, which would be an example of what Arnstein (1969) calls “tokenism” in his ladder of citizen participation. The ability to participate is another defining factor. O׳Connor et al. (2006) describe the development of video tools with a severely disabled person with no language and point to the difficulties in finding appropriate means of expression and in interpreting their limited communications from a empathic standpoint.

*How do stakeholders and participants benefit*? While often the main benefit for participants is believed to be provided by an improved or better design (see discussion on outcomes below), the direct impact on the participants themselves is often overlooked (notable exceptions include Clement and Besselaar, 1993, Balka, 2006). This motivated Bossen et al. (2010) to conduct a study into the self-reported gains of stakeholders from participating in a 5-year research project within an educational context. They interviewed pupils, teachers, administrative staff, consultants and one politician in order to assess their personal gains, understand their frustrating and satisfactory experiences, and consider perceived influence on the project and any impact on their future development. They found that participants gained on several levels, such as improved competence with technology, the awareness of novel educational opportunities and the building of relevant social networks. In a follow up study, Bossen et al. (2012) used the same methodology in a different context to investigate the main impediments on realising gains for participants. They found the most significant hurdles to be related to unresolved differences in aims, to ambiguities in the structure of the collaboration and to different conceptions of technology. Notably, they highlight the importance and value of such reflective studies to “*make it clearer how and in which way PD projects reach the goals they strive for.*”

*What happens when the project ends*? While not all PD projects may have the ambition to create a lasting legacy, most still share a notion of revealing alternatives or possibilities for change through participant involvement. In this sense, a PD effort may have a sustained impact in different ways, for example, through altered structures, practices, perspectives or technological opportunities within the world of participants. In their review of early PD projects, Clement and Besselaar (1993) found that many of the projects could be considered successful in facilitating the involvement of stakeholders, but none had translated into a self-sustained, local process of participation once the projects had ended. They argue that this would require participants to become local actors—“animators”—who take over the initiative and do something that is inspired by their experience of having been involved. We agree, and believe the question of sustainability in the context of PD is ultimately not a question of structures or politics, but one of enabling and motivating participants and turning them into advocates. Bossen et al. (2010) report that several of their participants voiced frustrations about the fact that initiatives faded within the institution after the project has ended. However, they also identified a number of indirect ways in which participants were able to bring experiences, skills or networks generated in the project to other contexts and thereby created a long-term impact of the work.

Exit strategies can be complicated and have an ethical dimension: while building relationships with participants is often carefully planned, ending such relationships hardly is. Depending on the nature of the collaboration, this can be a natural part of ending a project, or a emotionally difficult situation. Gary Mardsen, for example, has highlighted this issue in discussions at the “Participation and HCI” Special Interest Group meeting at the CHI׳12 conference. Referring to his work in rural areas of Africa he said “*When you spend so much time building a rapport with people it is very difficult to suddenly become removed from this social context and environment*” (Vines et al., 2013). Beyond the emotional aspect, participatory work may have created real dependencies where designers, researchers or practitioners have become an integral part of the change that the work has aimed to achieve.

### 3.4 Outcomes

Assessing the outcomes with respect to the impact participation has made, became the most important way to justify participatory approaches, particularly at the more pragmatic end of the PD spectrum. When external pressures influence the design process, such time and budget constraints, PD must answer questions about its effectiveness. We see two main challenges in making PD accountable in this respect: one is related to the definition of outcomes and the second is of an epistemological nature. Firstly, the diverse motivations behind PD and relatedly, the diverse groups of people involved, mean that what constitutes outcomes and how they are assessed depends on the perspective taken. Outputs could manifest themselves in changes to local practices or artefacts for the mass market, and both could be considered the main outcomes of the same PD effort depending on who one asks. Secondly, establishing the impact the participation of non-designers had on the outcomes is not trivial. The highly contextual nature of PD work makes it very hard to demonstrate the added benefit of participation *in comparison* to non-participatory approaches since comparative studies would be highly impractical.

While most commonly *the output* is associated with the actual artefact or design, there are many ways in which outcomes of PD projects may be perceived. For example, PD׳s history and shared background with Action Research highlights social change as a possible desired outcome (Foth and Axup, 2006). And as the section on stakeholders has argued above, direct impacts on people involved in the project also might be seen as outcomes of the project, whether by design or unintentional. Discussing co-design spaces, Sanders and Westerlund (2011) draw attention to the need for reflection on questions such as “*Who determines what the output means*? *What is the collective outcome*? *What is the individual outcome*?” highlighting the fact that these definitions of outcome may differ substantially depending on personal perspectives. Zimmerman et al. (2010) make another point when discussing the relationship between Design Research and science: in conducting research through design, which many PD projects do implicitly or explicitly, contributions can be made to both methodology and knowledge (see above).

Once outcomes have been identified, establishing whether participation has directly or indirectly benefited these outcomes is a further challenge. Irestig et al. (2004) report on the only comparative study we were able to find to directly investigate this. They developed information system prototypes in the context of a Swedish Trade Union project in two parallel research streams: one following a PD approach, one a user-centred design (UCD) approach. They then used an analytical framework originating in Activity Theory to systematically compare the outcomes, and were able to isolate certain differences in terms of the characteristics of the two designs. They found, for example, that the PD solution focused on collective activities and organisational practices, while the UCD solution focused on single user use and the adaptability of the system. While this might not be surprising given the foci of the different design methods employed, the study seems to confirm that the intended benefits of the PD method translate into actual qualities of the system, and are thus a consequence of participation.

In a broader scope, Kujala (2003) have conducted a literature review on the benefits of user involvement on system design. Their interpretation of *beneficial* is limited to what is beneficial to the qualities of the actual system—the artefact as the outcome—and the review does not focus on PD only, but includes any form of user involvement, e.g., UCD, contextual inquiry and ethnography. Reviewing field studies, qualitative research and quantitative work, they found support for several common claims, for example that user involvement increases the level of user acceptance, but evidence for others was not forthcoming, for example the cost-effectiveness of user involvement. They summarise tellingly that “*The effects of user involvement seem to be positive overall, but complicated*.”

As a consequence, we have omitted a starter question that directly probes for the impact of participation on outcomes. The nature of PD makes it either nearly impossible or trivial to assess a causal relationship between any outcomes and participation and in both cases such a question would not contribute to describing the level of rigour or increase accountability. We do, however, argue that using outcomes as a reflective perspective has its value, particularly in that outcomes are often the focus of attention when PD work is being held accountable. While this may not be fully justified, the following starter questions aim to redirect the focus towards features of outcomes that are more appropriate in the context of PD:

*What are the different interpretations of outcomes*? In a recent study, Garde and van der Voort (2012) asked participants of a PD project that aimed to support the design of a new hospital ward, to describe the main outcomes of the workshops. Anonymised responses were collected through a post-event questionnaires and subjected to content analysis. They found remarkable differences in what participants took away from the workshops depending on their roles or the particular activities. The results, they argue, illustrate the value of probing for interpretations of outcomes after PD activities, most importantly to re-adjust subsequent activities. Post-event questionnaires seem to be an effective way to do this, but the authors also state the danger of getting “socially pleasing” answers, so in some contexts other methods might be more appropriate. Notably, they included themselves as participants in the study too. Given that designers often make design decision on the basis of what they believe to be the agreed outcome of a PD activity, an awareness of the divergent interpretations of the outcomes adds important information to the basis for these decisions.

*Who owns outcomes*? Ownership of outcomes is a complex psychological and sometimes fluid state that many PD projects observe in the behaviour of participants, e.g., with respect to the interaction with an artefact or the role participants assume in an altered social environment. PD work on the idealistic side of the spectrum often makes this shift of ownership an explicit goal, while on the other side of the spectrum ownership is more likely to be retained by the designers. However, there are also more nuanced cases in which ownership is perceived as shared. In any case, ownership reflects features of the design process and the roles participants are given. Thus, it relates to the stakeholder lens, but while our concern in the stakeholder lens was mainly focused on relationships and decision making, the question of who owns outcomes adds another dimension to assessing benefits and motivations. For example, when van Rijn and Stappers (2008) developed LYNKX, a language learning tool for children with autism, they demonstrated that fostering ownership in the design process was a key factor in motivating participants to contribute. Merkel et al. (2004), in reflecting on three community projects in which they co-designed technology, describe this process as “*Seeding ownership*”, and it is a pre-requisite for designing for sustainability—which leads us to the final question in this lens:

*How sustainable are outcomes*? Above we argued that sustainable PD requires stakeholders who become advocates, “animators” (Clement and Besselaar, 1993) and owners (Merkel et al., 2004). In itself this qualifies as an outcome, but it is easy to see how design outcomes, such as services or artefacts, can themselves engender sustainable participation, for example, services which transform practices in the long run. In this sense, it is worth considering which outcomes have the potential to survive beyond the end of a PD project, and the qualities they must possess in order to do so.

## 4 Rigour, accountability and coherence

Rigour manifests itself in different ways, depending on the science paradigm one operates in Guba and Lincoln (1994). While in positivism and post-positivism rigour emphasises validity and reliability of results (compare Fallman and Stolterman, 2010), other fields have sought other criteria that fit their own belief system as we have discussed at various points above. The heterogeneity of PD makes establishing a set of universal criteria impossible. Too broad is the spectrum of interpretations of PD, too varied are the motivational drivers behind PD work. Similar to Fallman and Stolterman (2010) we argue that any notion of rigour has to be developed within a “*firm understanding of the particular purpose of each approach*.” Different PD projects might be equally rigorous in and by themselves, and still bear very little resemblance in terms of their processes, methodologies or outcomes.

Equally, accountability is not a label that confirms the presence of certain features, as in a chain of evidence, but the ability of designers, researchers, practitioners and indeed stakeholders to construct an argument for the appropriateness of the process and the trustworthiness of outcomes. As with rigour, such an argument can take many forms and depends not only on the context and one׳s interpretation of PD, but also on the intended audience. For example, being held accountable by a funding body, academic peers or effected community groups will require a different focus in argumentation. We thus emphasise that our “tool-to-think-with” provides a language for constructing such arguments, not the arguments themselves.

Guided reflection brings to the open the many tacit features that define a PD process, but by itself is insufficient to get a handle on rigour and accountability. Both concepts, rigour and accountability are at their core concerned about a notion of quality of PD work that accommodates for the variety of contexts, approaches and theoretical foundations, but signifies appropriateness, thoroughness and trustability. We therefore have developed the concept of *coherence*.

Analysing the *coherence* means analysing how well the features of a PD effort, revealed through guided reflection, are attuned to each other; whether the features are coherent in the sense that they pull the project in the same direction or work against each other. We argue that rigorous PD work exhibits qualities that are coherent, e.g., it is based on an *epistemology* that accommodates the *values* that drive the effort, involves *stakeholders* in ways that reflect these foundations and accordingly defines and delivers its meaningful *outcomes*.

Importantly, *coherence* does neither imply absolute agreement nor does it prescribe a single interpretation of “good” PD work. For example, PD work can exhibit internal rigour with many different, even conflicting values present when this is reflected in the way outcomes are defined and stakeholders are involved. And we would argue that a PD effort has equal internal rigour when the underlying epistemology is pragmatic, stakeholders are involved purposefully or selectively, and outcomes are defined with a clear focus on an artefact. The *coherence* of features neither makes judgments about the level of agreement within the lenses nor where on the spectrum of PD ideology the effort is defined. The level of *coherence* would be low, however, when, for example a PD effort is driven by democratisation, but leaves key stakeholders no scope for change. Or the epistemology is situated and the outcome is a mass product.

To further illustrate the concept we turn to two examples of what might be the result of applying our “tool-to-think-with” on aspects of the ECHOES project: The proposal to the funding body described an empirical study to assess the effectiveness of the system to be designed as the main outcome. The epistemological stance the study design implied made it necessary to build the system so that it could be evaluated along specific, measurable dimensions. The proposal also emphasised a participatory mindset in designing the system and thereby implicitly adopted a set of values that revolved around empowerment of children with autism. While this set of values might have been interpreted more radically by the members of the PD strand, there was also a sense that the fundamental orientation was shared across the whole project. Analysing the *coherence* of these aspects of the proposal makes clear that there were serious conflicts between the epistemology, the values, the outcomes and the kind of involvement of stakeholders, which, at the time went largely unnoticed. Firstly, the values around empowerment could not be followed through into the required outcome as the type of the outcome—the empirical evidence—required us to limit the impact children could have on the design process and the system itself. Secondly, the epistemology within which PD worked was not coherent with the epistemology the outcome required. While PD presented knowledge in a situated context that reflected their work with children, colleagues who were concerned about the evaluation extrapolated the epistemological framework of the empirical study and expected PD to deliver a different kind of knowledge. Looking through the stakeholder lens makes clear that the PD strand had ignored an important stakeholder: PD has not actively involved their own colleagues to resolve the epistemological incoherences. All this originated in the way the work structure of the project was set up. PD was made a parallel strand, separate from the design of learning activities, the technical realisation and the evaluation. Consequently, the incoherences turned into conflicts in the interfacing between those strands.

In the second example, we analyse the PD Critique activity in ECHOES (Frauenberger et al., 2013): while researchers in the PD strand had a particular outcome in mind before they conducted this series of activities, in the course of the pilot studies the outcomes were completely re-defined. The epistemological paradigm the researchers operated within allowed them to make this shift and to embrace the explorative nature of the resulting studies as they were not bound by other stakeholders to deliver a solution to a particular question. Within the value-system of the researchers involved it was clear that the new direction this activity took was much more valuable than the original aim. I.e., it was more valuable to gain a broad and nuanced understanding of supporting participation of children with difficulties in social communication than establishing the effectiveness of a tool in generating new design ideas. The shift led to situated, methodological knowledge and insight with respect to the nature of participation of this group. In this example the values were coherent with the epistemology which in turn allowed the researchers to pursue the adjusted outcomes through appropriate methods. We would therefore argue that this second example exhibits more internal rigour than the first.

We acknowledge that both examples are incomplete snapshots, meant to illustrate the concept rather than necessarily accurately portray rigour in ECHOES. However, they show how articulating reflections from particular perspectives and assessing their coherence allows one to make qualitative statements about PD work.

## 5 Operationalising the framework

There are three different ways in which we believe operationalising our approach can be beneficial to practitioners:

Firstly, our framework can support practitioners in setting up PD work by clarifying core starting points. Identifying required or desired features of the project through critical reflection provides the basis on which the practitioner can develop an appropriate methodology and consider the fundamental standpoint they take along the spectrum of PD approaches. Analysing the coherence between those qualities additionally supports them in identifying particular challenges that either require a methodological resolution or a critical review of aims. In the case of ECHOES using our framework in the planning phase would have revealed a number of fundamental problems, such as the incompatibility between epistemological stances discussed above. It undoubtedly would have flagged the incoherence between some of the PD aims and the decision to make PD a separate strand. As a consequence, a number of changes would have been possible without jeopardising the overall goals. PD could have been interwoven with other strands thus underpinning the outcomes rather than supplementing them. Different outcomes could have been defined to acknowledge the different scientific cultures and the different kinds of knowledge the work constructed. PD could have actively engaged other team members in their work to develop a mutual understanding for the kind of knowledge that is constructed. This would also have allowed a different timeline in which some PD work would have preceded other developments.

Secondly, our framework can be operationalised as part of a reflective practice. PD processes are inherently non-linear and reflect shifting intent and ongoing discovery as the second example from ECHOES in the previous section exemplified (compare also Wolf et al., 2006, on design practice). Adjustments to the process, just like design decisions themselves are the result of judgments rather than deductive reasoning (Löwgren and Stolterman, 2007, p. 54) and are informed by the practitioners׳ skills, expertise, values and context dependent information at the time. Many have pointed to critical reflection as a key resource for making judgments. Beginning with Schön (1983), researchers have built the case for reflection-in-action as a key aspect in ensuring quality and relevance in design. Sengers et al. (2005) have coined the term Reflective Design in HCI, stating that “*technology design practices should support both designers and users in ongoing critical reflection about technology and its relationship to human life*”. Drawing on Critical Theory and other related approaches (e.g., Bardzell et al., 2014), they derive principles and strategies for a reflective design practice. Bødker and Iversen (2002) argue that reflection in action and off-loop reflection are the defining features of expertise in PD, enabling practitioners to work in systematic ways, moving beyond trial and error and ad hoc design decisions. Thus, revealing the tacit qualities, which are a kind of knowledge-in-action (Schön, 1983), and identifying issues with coherence can help PD practitioners to inform their navigational judgements in a more transparent and systematic way, thus, in itself increasing the rigour of their work.

In inter-disciplinary projects like ECHOES, accountability also plays a key role in communicating PD and dealing with conflict. The language afforded by our framework enables designers, researchers and practitioners of PD to construct arguments that underpin their judgements and decisions. They are enabled to adjust these arguments to accommodate different peer groups, inside and outside the project.

Finally, like most of our statements about ECHOES in this article demonstrate, our framework affords a post hoc analysis of PD work. In a maturing field like PD, it is critical to instil a notion of trustworthiness in our efforts by the way of critical analysis. It is the key requirement to enable the field to build on findings and progress. As discussed above, PD knowledge can be transferred when processes and judgments are transparent and contextual dependencies are carefully considered. Our framework supports practitioners by making such information available. Secondly, to advance the field as a whole, it is important to be able to systematically scrutinise, critique and communicate work and we see our framework as a contribution to this end too.

## 6 Conclusions and future work

With this article we have proposed a “tool-to-think-with” that guides PD practitioners in a process of critical reflection on their work. The concept of coherence provides a robust yet flexible means of assessing and communicating internal rigour and accountability. Throughout we have sought to ground our thoughts through illustrative examples from a specific PD experience, ECHOES, that highlight some of the underlying challenges in conducting participatory work and communicating it to peers.

In future work, we envision our approach being developed further by applying it to other PD work and at various phases of projects. The lenses and their starter questions are likely to evolve with new aspects emerging from different contexts or from projects with particular foci. For example, in projects where vulnerable groups are involved, the stakeholder lens could include more specific questions regarding ethics and the outcomes lens would require more emphasis on interpretation and empathy. The framework is open for such tuning, but we argue that the fundamental concept of combining a structured, critical reflection with a meta-analysis of the coherence of the findings is an appropriate way of qualifying work in our field and hope this approach will find its way into the working practice of many PD practitioners. To further develop our framework we have plans to invite other researchers and practitioners to bring their PD experiences to workshops and we also plan using the framework in educational settings where it guides students in their learning of PD practices by supporting their critical self-assessment.

## Footnotes

1 Note: outcomes in this context is not restricted to technological artefacts, but refers more broadly to a desired alternative future.

2 ESRC/EPSRC, TRLP TEL programme grant number: RES-139-25-0395.

3 Paraphrased from its definition in the online Oxford Dictionary (http://oxforddictionaries.com/) as well as from Friedman et al. (2008) and Halloran et al. (2009).
